# Understanding and Tackling the Complex Challenges of Homelessness and Health

**DOI:** 10.3390/ijerph19063439

**Published:** 2022-03-15

**Authors:** Kate Frazer, Thilo Kroll

**Affiliations:** School of Nursing, Midwifery and Health Systems, University College Dublin, D04 V1W8 Dublin, Ireland; thilo.kroll@ucd.ie

Homelessness is a complex global public health challenge. There is no universally agreed definition of homelessness, and it may refer to individual rooflessness as well as to the forced displacement of populations. The European Federation of National Organisations Working with the Homeless (FEANTSA) devised the European Typology of Homelessness and Housing Exclusion (ETHOS), which distinguishes between rooflessness, houselessness, living in insecure housing, and living in inadequate housing, there is a dearth of reliable data on the prevalence of homelessness. As a result of the challenges in defining homelessness, data on the prevalence of the phenomenon vary. Moreover, resources and logistics to assess the extent of homelessness diverge among countries globally. Estimates suggest that one percent of the global population is affected by homelessness [1].

People of all ages and families become homeless for a variety of reasons. Natural or human-made disasters, war, family breakup, forced migration, lack of affordable and adequate housing, loss of employment or a combination of these factors.

There is considerable evidence that people with existing health problems, especially those with untreated mental health problems or inadequate access to health care services, are at greater risk of becoming homeless at some point in their lives. The literature is clear in that the life expectancy of people who have homelessness experiences is lower than that of the general population. We also know that health risk behaviours such as drug, nicotine and alcohol dependency are substantially higher than for people who are not homeless. Suicidality rates are substantially higher in the homeless population. Just as poor health can be one of the risk factors for losing one’s home, physical and mental health concerns are considerably more prevalent in the homeless compared to the general population. In other words, chronic homelessness is associated with a range of negative health outcomes and disabilities.

The experiences of people who live in displaced person camps may be very different from individuals facing urban homelessness. However, what they have in common is the precariousness of their circumstances, the loss of a sense of belongingness and uncertainty of the future.

Being homeless is associated with a plethora of challenges related to the environment that negatively impact health and functioning. Those managing chronic conditions in the context of homelessness are more likely to seek care in emergency departments and have more hospitalisations than people who are not homeless. Access to timely, appropriate and coordinated care is a particular problem for many. Shelter conditions may not be conducive for managing long-term conditions. Increasingly, the impact of adverse weather events and environmental hazards is considered in relation to health and homelessness.

There have been several approaches to tackle homelessness at the systems level. Housing First initiatives place the housing issue at the centre [2]. However, many well-intended strategies and interventions fail, either by their inability to follow a person-or family-centred approach or by not providing agile and adaptable and integrated support to individuals and families over time as economic, social, health-care and educational needs evolve.

This Special Issue considering health and homelessness coincided with the COVID-19 pandemic and includes research highlighting its impact. The preparation and submission of manuscripts over this period of time represented challenges to authors themselves, and we acknowledge their support and the journal in extending the submission period. In this editorial, we summarise the contents of the special collection and consider future research.

The research includes contributions from the United States, Ireland, Australia, Canada, Brazil, England, Scotland and Spain. Two papers report concurrent challenges due to COVID-19. Parkes [3] provides insight into the impact of COVID-19 and considers a harm reduction alcohol management programme; Nunes [4] highlights the impact of Third Sector organisations in supporting socially and emotionally.

The health impacts of homelessness presented by Eshtehardi [5] examine prescription management in supporting a comorbid diagnosis of depression, Carroll [6] reports an absence of access to rehabilitation services, Vallesi [7] identifies tri- and multi-morbidity, identifying the pervasiveness of preventable health conditions that exist for this population and limited access to screening programmes, and Salem [8] examines the increased risk of latent tuberculosis infection. Broderick [9] presents the outcomes of a review of physical health screening tests and confirms the challenge in using unsuitable standard tests which add to increased pain and discomfort. Further harm reduction is considered by Durazo’s study [10], demonstrating the use of e-cigarettes in support of tobacco control.

Three studies provide a broader lens on homelessness. Nourazari [11] uses a systems approach to consider policy implications, and Calvo [12] presents a 10-year review of mortality rates and risk factors. Magwood [13] examines introducing clinical guidelines supporting health equity. This study embeds practitioner responses and those of people accessing services. Warren [14] presents the voices of people who engaged with a newly established nurse-led support service. Finally, two reviews are included. Murray [15] presents a lack of evidence of suicide-specific preventions, and Bezgrebelna conducts an umbrella review [16] of evidence of the impact of weather extremes and the impact for those disproportionately impacted.. 

The articles in this Special Issue provide an array of innovative research, insights and perspectives from a range of disciplines and perspectives, all of which present extensive challenging experiences, and many studies were not supported by any grant or external funding. We would have liked to have seen evidence from wider groups impacted by homelessness, including families, children and younger aged youths, ethnic minorities, displaced populations, and including a patient and public involvement approach.

Pleace [17] confirmed the initial gains and continued challenges that exist for the homeless sector in a post-COVID 19 Europe, noting the longstanding tendency to explain homelessness in terms of individual pathology: the choices, actions, needs, characteristics, and experiences of each individual; homelessness as a matter of ‘sin’ and ‘sickness’ rather than ‘systems’ [p.58].

We hope readers use the evidence presented, and we will be issuing a call for a new Special Issue considering a wider systems lens.

## Data Availability

Not applicable.
